# Human gut, breast, and oral microbiome in breast cancer: A systematic review and meta-analysis

**DOI:** 10.3389/fonc.2023.1144021

**Published:** 2023-03-17

**Authors:** May Soe Thu, Korn Chotirosniramit, Tanawin Nopsopon, Nattiya Hirankarn, Krit Pongpirul

**Affiliations:** ^1^ Joint Chulalongkorn University - University of Liverpool Ph.D. Programme in Biomedical Sciences and Biotechnology, Faculty of Medicine, Chulalongkorn University, Bangkok, Thailand; ^2^ Center of Excellence in Immunology and Immune-Mediated Diseases, Department of Microbiology, Faculty of Medicine, Chulalongkorn University, Bangkok, Thailand; ^3^ Department of Infection Biology and Microbiomes, Faculty of Health and Life Science, University of Liverpool, Liverpool, United Kingdom; ^4^ School of Global Health, Faculty of Medicine, Chulalongkorn University, Bangkok, Thailand; ^5^ Department of Preventive and Social Medicine, Faculty of Medicine, Chulalongkorn University, Bangkok, Thailand; ^6^ Brigham and Women’s Hospital, Harvard Medical School, Harvard University, Boston, MA, United States; ^7^ Department of International Health, Johns Hopkins Bloomberg School of Public Health, Baltimore, MD, United States; ^8^ Clinical Research Center, Bumrungrad International Hospital, Bangkok, Thailand

**Keywords:** breast cancer, dysbiosis, gut microbiome, microbial diversity, estrogen

## Abstract

**Introduction:**

Dysbiosis characterises breast cancer through direct or indirect interference in a variety of biological pathways; therefore, specific microbial patterns and diversity may be a biomarker for the diagnosis and prognosis of breast cancer. However, there is still much to determine about the complex interplay of the gut microbiome and breast cancer.

**Objective:**

This study aims to evaluate microbial alteration in breast cancer patients compared with control subjects, to explore intestine microbial modification from a range of different breast cancer treatments, and to identify the impact of microbiome patterns on the same treatment-receiving breast cancer patients.

**Methods:**

A literature search was conducted using electronic databases such as PubMed, Embase, and the CENTRAL databases up to April 2021. The search was limited to adult women with breast cancer and the English language. The results were synthesised qualitatively and quantitatively using random-effects meta-analysis.

**Results:**

A total of 33 articles from 32 studies were included in the review, representing 19 case-control, eight cohorts, and five nonrandomised intervention researches. The gut and breast bacterial species were elevated in the cases of breast tumours, a significant increase in *Methylobacterium radiotolerans* (*p* = 0.015), in compared with healthy breast tissue. Meta-analysis of different α-diversity indexes such as Shannon index (*p* = 0.0005), observed species (*p* = 0.006), and faint’s phylogenetic diversity (*p* < 0.00001) revealed the low intestinal microbial diversity in patients with breast cancer. The microbiota abundance pattern was identified in different sample types, detection methods, menopausal status, nationality, obesity, sleep quality, and several interventions using qualitative analysis.

**Conclusions:**

This systematic review elucidates the complex network of the microbiome, breast cancer, and therapeutic options, with the objective of providing a link for stronger research studies and towards personalised medicine to improve their quality of life.

## 1 Introduction

Breast cancer has been a major concern for women for several decades, with an estimated 2.3 million cases in 2020 and a global frequency of 11.7% (1). To date, four subtypes of invasive breast cancer have been identified: luminal subtype A, which exhibits high levels of estrogen receptor (ER) and progesterone receptor (PR), but low expression of human epidermal growth factor receptor 2 (HER2) and cell proliferation index; luminal subtype B, which exhibits ER/PR+, HER2, and high proliferation index; HER2+ breast cancer subtype; and triple-negative breast cancer (TNBC) subtype (2). The likelihood of breast cancer is typically increased by several risk factors, namely, age, sex, obesity, family history, genetic mutation, estrogen level, and sedentary lifestyles (3–5). The human microbiota, on the other hand, has drawn considerable interest as a key risk modulator due to its unique function in controlling steroid hormone metabolism by activating various enzymes, including hydroxysteroid dehydrogenase (6, 7).

The gut microbiota is an abundant ecosystem of highly diversified microorganisms, with the Firmicutes and Bacteroidetes phyla accounting for approximately 90% of the gut microbiota, including *Lactobacillus*, *Clostridium*, *Enterococcus*, *Dialister* and *Ruminicoccus* of Firmicutes and *Bacteroides*, *Alistipes*, and *Prevotella* of Bacteroidetes (8). An imbalance of the human gut microbiota known as dysbiosis causes a number of health problems (9–11). A comparison of many breast samples reveals differences in the quantity and microbial diversity of several specific genera between healthy people and patients, despite the absence of conclusive evidence that dysbiosis causes breast cancer (12, 13). It also acknowledges the relationship between different gut microbial profiles and different subtypes of breast cancer (14). Furthermore, although there was no significant variation in abundance between premenopausal breast cancer patients and controls, the structure and functions of the gut microbial community differed between postmenopausal breast cancer patients and healthy controls (15). As a result, the impact of microbial instability in the breast, as well as the function of microbial communities in the development of breast cancers, has been thoroughly established.

Tumour tissue and high-risk tissue had a much lower breast microbial diversity than tumour-neighbouring normal or healthy control tissue adjacent to the tumour. For example, the breast tumour microbiome contained a higher proportion of the Pseudomonadaceae and Enterobacteriaceae families, the genera *Pseudomonas*, *Proteus*, *Porphyromonas*, and *Azomonas*, compared with other tissues (16). On the other hand, propionibacterium and Staphylococcus were rare in tumour tissue but were important components of healthy control, high-risk, and neighbouring normal tissues (16).

Breast cancer and the oral microbiota, in particular, appear to be linked. The risk of breast cancer has been found to be higher in women who have periodontal disease, caused by specific bacteria such as the red complex (*Porphyromonas gingivalis*, *Tannerella forsythia*, and *Treponema denticola*) and the orange complex (*Fusobacterium nucleatum*, *Prevotella intermedia*, *Prevotella nigrescens*, *Peptostreptococcus micros*, *Streptococcus constellatus*, *Eubacterium nodatum*, *Campylobacter showae*, *Campylobacter gracilis*, and *Campylobacter rectus*) (17–19).

The gut microbiota secretes bioactive bacterial metabolites, such as reactivated estrogens, amino acid metabolites, short-chain fatty acids (SCFAs), or secondary bile acids (BAs), which can affect disease progression (20–22). For its estrogen reactivation activity, the role of gut microbial β-glucuronidase (GUS) in the pathogenesis of breast cancer has been proposed, and GUS is encoded by Bacteroidetes and Firmicutes in the human gastrointestinal tract (23). Glutamine-proline-glycine metabolism became active in different subtypes of breast cancer, and amino acid transporter-2 metabolites were up-regulated to serve energy homeostasis and protein and nucleotide biosynthesis (22). SCFAs are essential in cell homeostasis, affecting the colon and other organs through blood flow, and are produced by two major bacterial groups: Bacteroidetes produce propionate and acetate, while Firmicutes produce butyrate (24). BAs, which are soluble derivatives of cholesterol produced in the liver, were previously thought to be carcinogenic agents but can have antineoplastic properties in cases of breast cancer (25).

As a result, the impact of the gut microbiome is multifaceted and important in controlling the host immune system in the pathophysiology of the development of breast cancer and the response and resistance to various cancer therapies (26–28). Through studies in animals, chemotherapy was found to alter the intestinal flora, which can result in adverse effects from early breast cancer treatment including weight gain or neurological disorders (29). Experimental research also showed a link between the gut microbiota and clinical outcomes and therapeutic responsiveness in different subtypes of breast cancer (30, 31). In particular, a study finds that gut bacteria are significantly more prevalent in breast cancer patients than in healthy people, which is detrimental to the prognosis of the disease (29). Thus, for therapeutic purposes and for the prognosis of the disease, detailed insights into the breast cancer oncobiome are important.

In recent decades, numerous studies revealed the impact of the microbiome on different organ-specific cancers and the action of bacterial metabolites in the human host on several signaling pathways, for example, E-cadherin/β-catenin pathway, breaking DNA double strands, promoting apoptosis, and altering cell differentiation (32–34). In particular, there are still several questions between the human microbiome and breast cancer development: “What pattern of the microbiome profile do breast cancer patients have in contrast to nonbreast cancer subjects”; “How different treatments modify the microbiome”; and “What is the microbiome profile in the same treatment”. To address these, a systematic literature review and meta-analysis on breast cancer and microbiome are conducted and the specific objectives are to evaluate microbiota alteration in breast cancer patients compared with nonbreast cancer subjects, to explore microbiota modification from a range of different treatment strategies, and to identify the impact of microbial pattern on the same treatment-receiving breast cancer patients.

## 2 Materials and methods

### 2.1 Protocol and registration

The systematic review of the literature was registered on PROSPERO ID 2021 CRD42021288186.

### 2.2 Literature search

The PRISMA statement guidelines were followed to conduct systematic review and meta-analysis (35). The study search strategy was developed based on the PICO/PECO (Population, Intervention/Exposure, Comparison or Controls, and Outcome) framework (36, 37). Two authors (MT and KC) independently examined each study for inclusion in the systematic review using PubMed (https://pubmed.ncbi.nlm.nih.gov/), Embase (www.embase.com), and the Cochrane Library (www.cochranelibrary.com). This was conducted using a full search term strategy, as detailed in **Supplementary Table S1**. The search was limited to adult women with breast cancer and the English language. Studies that included only nonhuman subjects or were not peer reviewed were excluded. Both epidemiological and intervention studies were considered from these databases and mainly focused on the interlink between breast cancer patients and the gut microbiome that was being extracted up to April 2022. Discrepancies were resolved by group discussion at each step.

### 2.3 Study selection

Article selection was carried out by two independent reviewers (MT and KC) for eligible studies using prespecified inclusion and exclusion criteria, followed by the full text review process. All relevant full-text articles were taken for further data extraction. The inclusion criteria for the meta-analysis were established as follows (1): Epidemiologic studies on how the microbiome profile in breast cancer patients differed from the pattern in nonbreast cancer control and (2) intervention studies on how treatment in breast cancer patients affected the microbiome and vice versa. Exclusion was performed in (1) the studies such as animal studies, *in vitro*, review articles, non-peer-reviewed articles, protocols, letters, editorial, commentary, recommendations, and guidelines and (2) the studies on breast cancer survivors. Disagreements between review authors were resolved by consensus at every phase of the selection of the systematic review selection.

### 2.4 Data extraction

The two independent authors (MT and KC) performed the data extraction for the following variables: (1) authors, year of publication, study period, study type, and country that implemented the study; (2) demographic characteristics such as menopause, menarche, and hormonal status; (3) related characteristics, including cytokine levels and enzyme activities; and (4) Parameters for the diversity profile. All relevant text, tables, and figures were examined during data extraction, and discrepancies between the two authors were resolved by discussion or consensus.

### 2.5 Risk of bias

The two independent authors (MT and KC) evaluated the risk of bias (ROB) in the extracted intervention studies. However, studies are nonrandomised trials, and therefore ROBINS-I (Risk Of Bias in Nonrandomised Studies—of Interventions) tool was applied to assess ROB (38). For included cohort and case-control studies, the two independent authors (MT and KC) performed the ROB evaluation using the Newcastle–Ottawa Quality Assessment Scale (NOS) developed from an ongoing collaboration between the Universities of Newcastle, Australia, and Ottawa, Canada, for quality assessment in a meta-analysis (39). The tool was used to assess the following domains: bias arising from the selection process, bias arising from the comparability process, and bias arising from the outcome/exposure process. Any disagreement was resolved by consensus. If there was not enough information to consider, the corresponding authors were emailed and their response was waited for 2 weeks. In the event of no response, it proceeded with the available data and any disagreement was resolved through discussion.

### 2.6 Statistical analysis

For intervention studies, mean differences (MDs) and a 95% confidence interval (95% CI) between groups were indicated for microbiome diversity outcomes. The characteristics of the participants, the study period, the type of study, and the location of the study were evaluated for clinical and methodological heterogeneity. The *I^2^* statistics were used for the assessment of statistical heterogeneity (40). The heterogeneity level was as defined in Chapter 9 of the Cochrane Handbook for Systematic Reviews of Interventions. For clinical, methodological, and statistical heterogeneity, the random effects meta-analysis using the DerSimonian and Laird method was utilized by RevMan 5, v.5.4.1 (https://training.cochrane.org/online-learning/core-software/revman/; accessed 31 October 2022).

## 3 Results

### 3.1 Study selection

**Figure 1:**
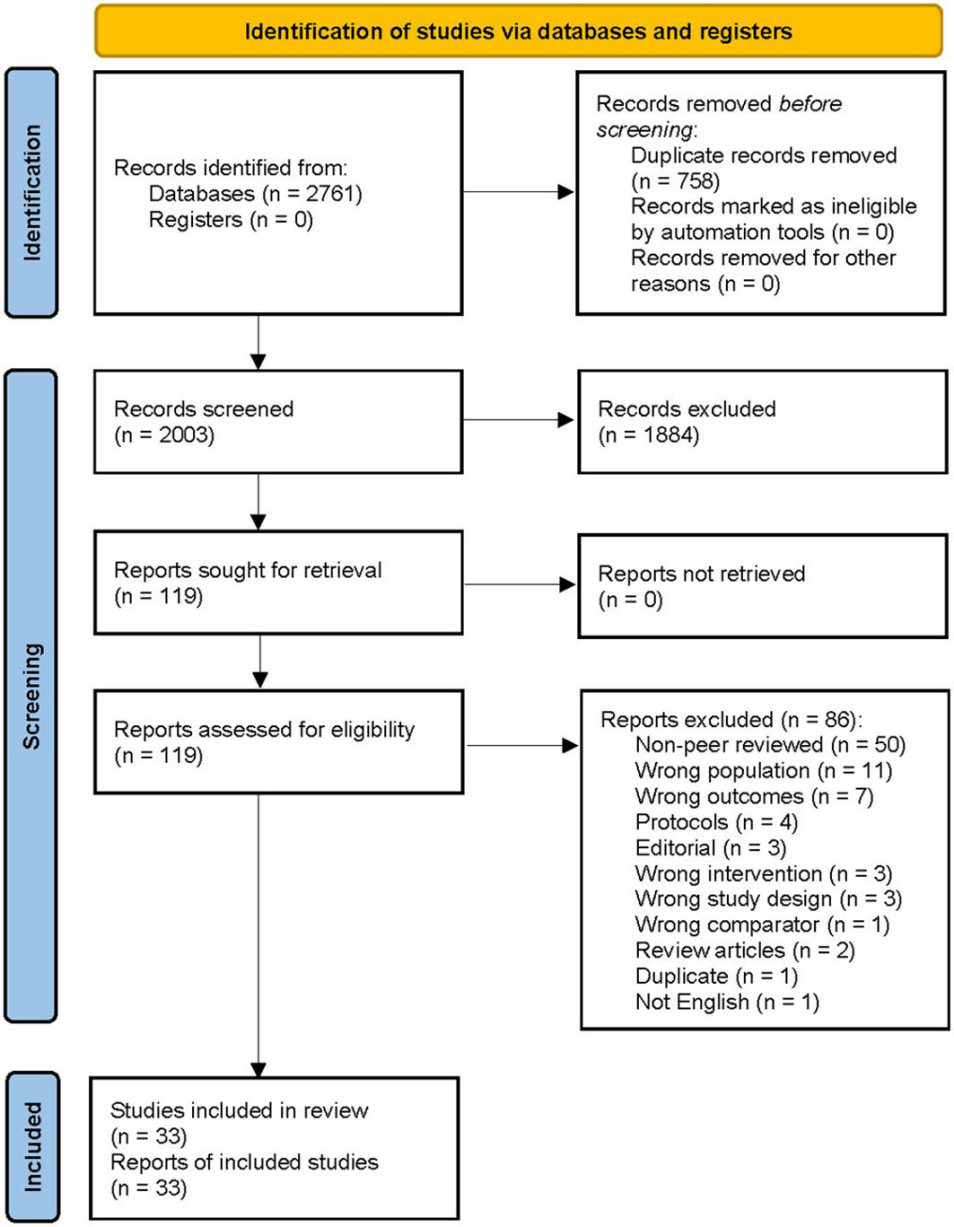
Flow diagram of the study selection in the systematic literature review.

The literature search found 2,761 articles from the databases, of which 758 duplicates were removed prior to selection. From the initial 2,003 studies, the title and abstract selection were carried out and 1,884 articles were excluded according to the inclusion and exclusion criteria. Next, we retrieved 119 articles for full text screening and checked their eligibility for meta-analysis. Among them, 86 studies were excluded due to the following conditions: 50 studies were articles not peer reviewed, 11 targeted the wrong population, seven raised wrong outcomes, four were protocol papers, three were editorial, three were wrong interventions, three were wrong study design, one were wrong comparator, two were review articles, one was duplicate, and one was not reported in English. Last, 33 articles from 32 studies, with an enrolment of 3,448 participants covering the study period from 2004 to 2019, were included in the systematic review and meta-analysis of the literature, representing 19 case-control, eight cohort, and five non-randomised intervention studies (**Figure 1**).

### 3.2 Study characteristics

The extracted studies were published from 1990 to 2020 in 11 countries, contributed mainly by the United States and China. We included different types of study, such as cohort, case-control, and intervention studies, and the age range participated was 18 to 90 years (**Table 1**).

**Table 1:** Baseline characteristics of the included studies.

First author	Published year	Study period	Study type	Country	Participants, n	Age range (year)	Ref.
Minelli	1990	–	Case-control	Italy	48	25-52	(41)
Benini	1992	–	Case-control	Italy	73	25-52	(42)
Xuan	2014	–	Case-control	USA	20	–	(43)
Urbaniak	2014	2012	Case-control	Canada and Ireland	81	18-90	(13)
Goedert	2015	–	Case-control	USA	96	50-74	(44)
Goedert	2018						(45)
Banerjee	2015	–	Case-control	USA	137	–	(46)
Urbaniak	2016	–	Case-control	Canada	71	19-90	(47)
Hieken	2016	–	Case-control	USA	33	33-84	(48)
Wang	2017	2014-2016	Case-control	USA	78	–	(17)
Thompson	2017	–	Case-control	USA	740	–	(18)
Huang	2018	2006-2015	Case-control	Taiwan	5	–	(19)
Banerjee	2018	–	Case-control	USA	168		(14)
Zhu	2018	–	Case-control	China	133	–	(15)
Smith	2019	–	Case-control	USA	83	18-72	(49)
Ma	2020	2017-2018	Case-control	China	50	–	(50)
Klann	2020	–	Case-control	Switzerland	46	–	(51)
UzanYulzari	2020	–	Case-control	Israel	33	18-75	(52)
He	2021	2019	Case-control	China	82	18-49	(53)
Byrd	2021	–	Case-control	Ghana	895	18-74	(54)
Luu	2017	–	Cohort	France	31	39.6–79.3	(55)
Meng	2018	–	Cohort	China	94	29-77	(56)
Costantini	2018	–	Cohort	Italy	16	46-82	(57)
Shi	2019	2017	Cohort	China	80	<45, 45-59, ≥60	(58)
Yoon	2019	2016-2017	Cohort	Korea	121	32-78	(59)
Thyagarajan	2020	–	Cohort	USA	23	27-78	(60)
DiModica	2021	2017-2019	Cohort	Italy	24	–	(61)
Yao	2020	2019	Cohort	China	36	–	(62)
Napeñas	2010	2004-2006	Non-randomized trials	USA	9	33-69	(63)
Frugé	2020	2014-2017	Non-randomized trials	USA	32	–	(64)
Chiba	2020	2004-2014	Non-randomized trials	USA	42	–	(65)
Wu	2020	–	Non-randomized trials	USA	37		(66)
Guan	2020	–	Non-randomized trials	China	31	36-66	(67)

### 3.3 Characteristics of the subject

Overall, the combined mean of the age of the participant is 54.3, with a standard deviation of 5.4. Regarding the menopausal status of the participants, there are three main groups: premenopausal, perimenopausal, and postmenopausal subjects. Among 66.4% of breast cancer cases, patients with postmenopause are 46% of the cancer patients are ≥ 13 years of age at menarche. In the study, 53.6% of breast cancer patients are from the United States, followed by Ghana with 16% and China with 14.6% (**Table 2**).

**Table 2:** Demographic characteristics of participants.

Characteristics	Breast cancer	Benign	Healthy
Participants, *n*	2362	238	868
Age (*M* ± *SD*)	54.3 (5.4)		
Menopausal status, *n* (%)			
Pre-menopausal	200 (32.5%)	13 (44.8%)	90 (51.7%)
Peri-menopausal	7 (1%)	8 (27.6)	0 (0)
Post-menopausal	409 (66.4%)	8 (27.6)	84 (48.3)
Menarche, *n* (%)			
Age ≤ 11 years	11 (30)	0 (0)	0 (0)
Age 12 years	9 (24)	0 (0)	0 (0)
Age ≥ 13 years	17 (46)	0 (0)	0 (0)
Countries, *n* (%)			
USA	1217 (53.6)	44 (18.5)	209 (24.1)
Ghana	379 (16.0)	102 (42.9)	414 (47.7)
China	344 (14.6)	63 (26.5)	99 (11.4)
Korea	121 (5.1)	0 (0)	0 (0)
Italy	86 (3.6)	0 (0)	75 (8.6)
Canada	72 (2.6)	24 (10.1)	28 (3.2)
Ireland	33 (1.4)	0 (0)	5 (0.6)
France	31 (1.3)	0 (0)	0 (0)
Israel	28 (1.2)	5 (2.1)	0 (0)
Switzerland	10 (0.4)	0 (0)	36 (4.1)
Taiwan	3 (0.1)	0 (0)	2 (0.2)

### 3.4 Risk of bias

**Figure 2:**
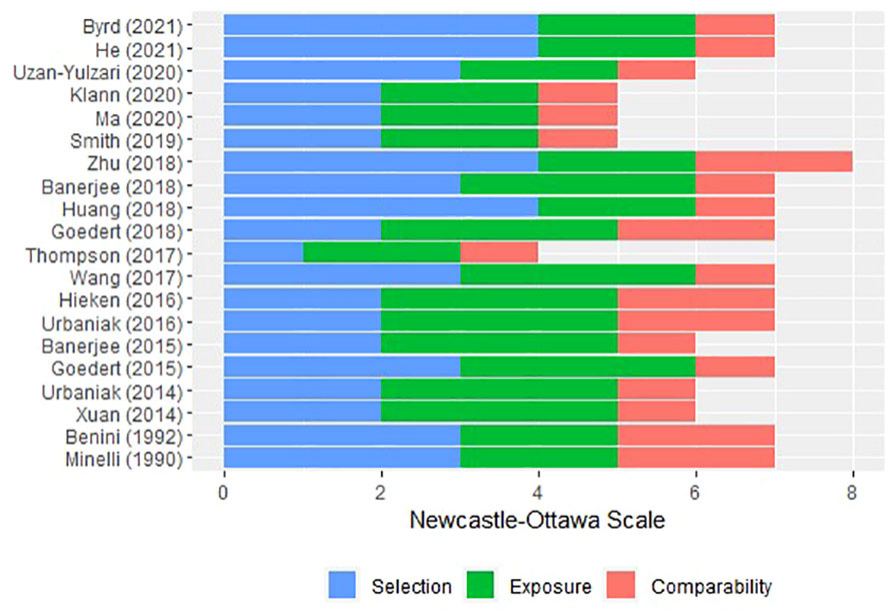
Risk of bias of the included case-control studies *via* Newcastle–Ottawa scale.

The ROB of the included studies was summarised by the study design group. ROB in case-control studies was evaluated mainly on selection, comparability, and exposure (**Figure 2**). The recruitment of subjects for 10 researches (14, 15, 17, 19, 41, 42, 50, 52–54) involved independent validation, while the subjects for the remaining research (13, 18, 43–49, 51) were often collected through databases or archival medical records. Four studies had the potential for selection biases: two (41, 42) did not disclose the selection procedure, one (18) used a genomics data repository to find patients but did not make a clear selection statement, and one (50) employed subjects from an Army-related hospital. Although control individuals from six trials (15, 19, 41, 42, 53, 54) were recruited from the community, the majority of control subjects was obtained as hospital controls, such as women who underwent breast reductions or cosmetic surgeries. Amongst them, two studies (48, 50) used mild cases as controls and one study (52) put the other cases of malignancy as controls. All cases and control subjects had comparability according to the study design, and the additional comparability measures were the same Mediterranean diet, age, and hormonal status by menopause. The exposure of the studies (13, 14, 17, 43–48) was identified by surgical records, while the other studies (15, 18, 19, 41, 42, 49–51, 53, 54) used written medical records. The same method of ascertainment and the same rate for both groups followed all the selection of subjects.

**Figure 3:**
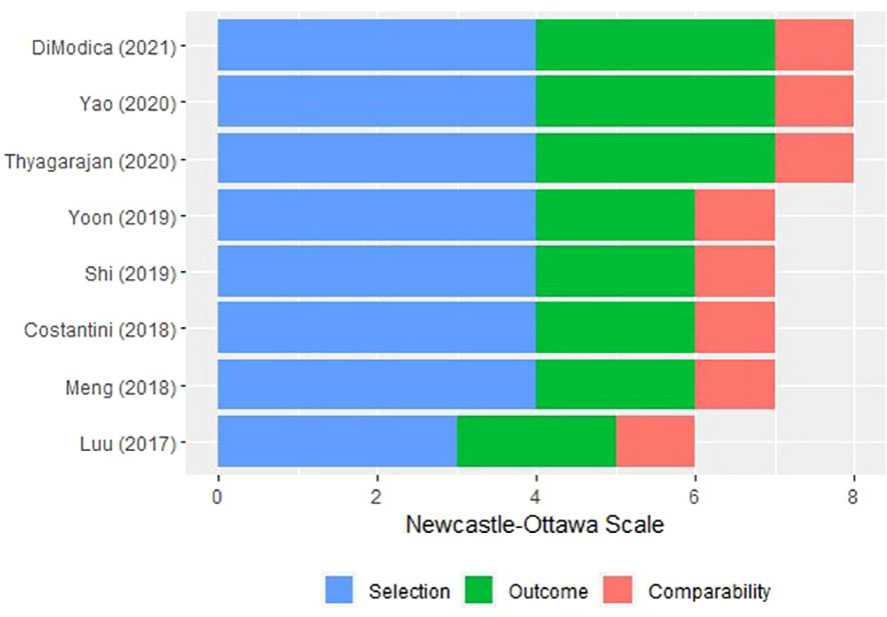
Risk of bias of the included cohort studies *via* Newcastle–Ottawa scale.

The selection, comparability, and ROB result in eight cohort studies (55–62) were also evaluated (**Figure 3**). A study (55) recruited participants from a volunteer group that was not representative of the community, but the other exposed cohorts (56, 57, 59–61) were recruited from nonmastectomy breast surgeries, patients planning breast cancer surgeries, or biopsy−confirmed breast cancer patients. All cohort studies had comparable study controls based on study designs and used medical records prior to outcome analysis. Probably there was sufficient time for follow-up; however, one still needs to outline a claim for the sufficiency. In a cohort study (60), some participants with admixed ancestry were excluded from the final analysis to reduce bias. No statement was found in other studies (55–59) on the suitability of follow-up.

**Figure 4:**
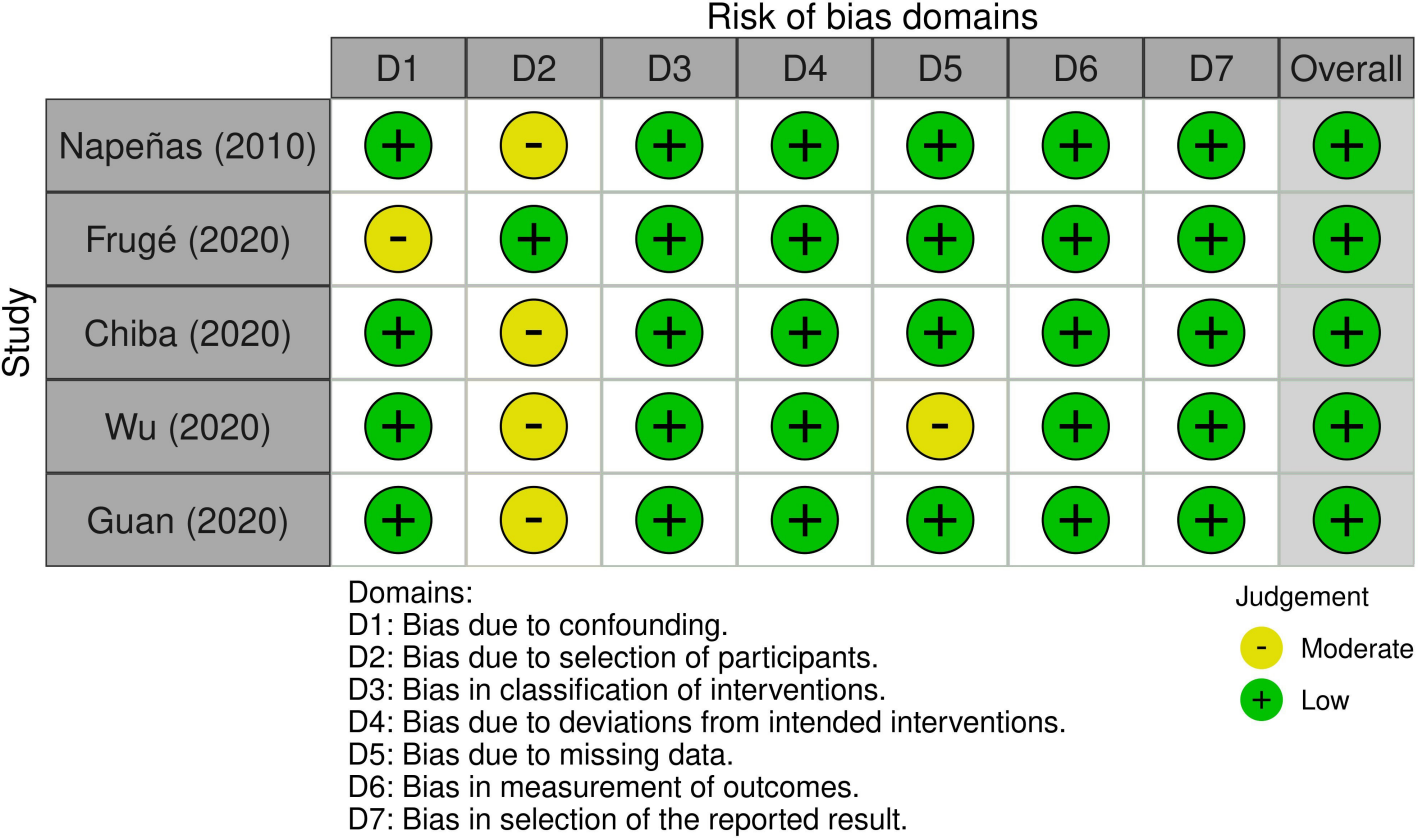
Risk of bias assessment for non-randomized intervention studies.

Most research domains were classified as having a low ROB in the ROB assessment of nonrandomized intervention trials (63–67) (**Figure 4**). Among them, four studies omitted details about the participant selection procedure and a confounding bias existed in one study (64). Another study (66) showed a tendency towards interventions that were not intended.

### 3.5 Qualitative analysis

Due to various sampling locations, different sequencing approaches, and various geographical and biological conditions, there was generally a lot of qualitative data when analysing microbial communities from different research. As a result, case-control, cohort, and non-randomised intervention studies were used to qualitatively analyse all the extracted studies (**Tables 3**, **4**), taking into account the study period, sample information, mean population age, microbial detection methods, microbiome type, and profile of microbial diversity.

**Table 3:** Characteristics of cohort studies and their microbial profiling and diversity.

Author/year	Study period	Sample	Age (Mean ± *SD*)	Microbiota and detection method	Bacterial profile and diversity	Ref.
Luu (2017)	NA	34 faeces samples of BC patients	62.3	Gut microbiota by real-time qPCR	According to the patient’s BMI, the absolute counts of total bacteria and three bacterial groups (Firmicutes, *Faecalibacterium prausnitzii*, and *Blautia*) vary significantly. Overweight and obese patients *vs.* normal BMI patients: ↑ total *Firmicutes, F. prausnitzii, Blautia* sp., and *E. lenta* bacteria, also relatively ↓ *F. prausnitzii.* According to the clinical phases and histoprognostic grades, the percentage and absolute counts of specific bacterial species, such as *C. coccoides, F. prausnitzii*, and *Blautia*, changed significantly.	(55)
Shi (2019)	2017	Faeces samples of 80 BC patients	NA	Gut microbiota by Illumina Sequencing	The patients were divided into 3 groups (TIL-H, TIL-M, and TIL-L) based on the levels of tumour−infiltrating lymphocytes (TILs). When comparing the TIL-L and TIL-H groups, as well as when comparing all groups, the β-diversity distribution was statistically significant. (*p* < 0.01) (Genus level) TIL-L *vs.* TIL-H: ↑ *Mycobacterium, Rhodococcus, Catenibacterium, Bulleidia, Anaerofilum, Sneathia, Devosia* and TG5, but ↓ *Methanosphaera* and *Anaerobiospirillum*. (*p* < 0.05) (Species level) TIL-L *vs.* TIL-H: ↑ *stercoris, barnesiae, coprophilus, flavefaciens* and C21_c20 species, whereas ↓ *producta* and *komagatae*. (*p* < 0.05)	(58)
Yoon (2019)	2016–2017	Faeces samples of 121 BC patients	50.26 ± 9.09	Gut microbiota by Illumina Sequencing	*Enterobacter* of Enterobacteriaceae: ↑relative abundance in high Fluorine-18-fluorodeoxy-glucose (18F-FDG) intestinal uptake (IU) group compared with low IU group (*p* < 0.001). Unclassified *Ruminococcaceae* trended towards being in lower relative abundance in the high IU group compared with the low group (*p* < 0.001). No statistical significant difference in α-diversity and β-diversity (*p* = 0.102) of gut microbial taxa between the lower and higher IU groups.	(59)
DiModica (2021)	2017–2019	Faeces samples of 24 BC patients	56.33	Gut microbiota by Illumina Sequencing	Nonresponsive patients had decreased α-diversity and abundance of *Lachnospiraceae, Turicibacteriaceae, Bifidobacteriaceae*, and *Prevotellaceae* compared with those who had a complete pathological response.	(61)
Yao (2020)	2019	Faeces samples of 36 BC patients	46.95 ± 9.87 *vs.* 49.18 ± 5.82	Gut microbiota by Illumina Sequencing	Phylum level: Women with poor sleep quality had ↑ Firmicutes (*p* = 0.021) and ↓ Bacteroidetes. Enterobacteriaceae was much higher in the no-sleep disturbance group. (*p* = 0.028) Genus level: Women with poor sleep quality harboured ↑ Acidaminococcus and ↓ genera such as *Alloprevotella, Desulfovibrio, Lachnospiraceae*_UCG-003, *Paraprevotella, Anaerotruncus, Prevotella*_2, and *Tyzzerella*_4. *Alloprevotella*: Adversely related to peak pain during movement within the first 24 hours. (*r* = 0.592, *p* = 0.001) *Desulfovibrio*: Anxiety symptoms are adversely linked. (*r* = 0.448, *p* = 0.006) Faecal microbiota richness: ↓ as the sleep quality deteriorated. No difference in α-diversity between the two groups. A substantial difference between the two groups was discovered using PERMANOVA (*p* = 0.02).	(62)
Meng (2018)	NA	Needle biopsies from 72 BC patients and 22 benign patients	52	Breast microbiota by Illumina Sequencing	*Propionicimonas* and Micrococcaceae, Caulobacteraceae, Rhodobacteraceae, Nocardioidaceae, and Methylobacteriaceae, which appeared to be ethnic-specific, were among the enhanced microbial biomarkers in cancerous tissue. With the progression of malignancy, the Bacteroidaceae declined, and that of *Agrococcus* increased.	
Costantini (2018)	NA	Core Needle Biopsy (CNBs) and Surgical Excision Biopsy (SEBs) from 16 BC patients	59	Breast microbiota by Ion PGM Sequencing	The OTUs provided 4 phyla (Proteobacteria, Firmicutes, Bacteroidetes, and Actinobacteria). *Ralstonia, Methylobacterium*, and *Sphingomonas* accounted for roughly 50–75% of relative abundances. The *Staphylococcus* and *Pseudomonas* and the Bradyrhizobiaceae and Rhodocyclaceae families accounted for 25–50% of the total.	(57)
Thyagarajan (2020)	NA	Fresh frozen tissue of 23 BC patients, including 13 White non-Hispanic (WNH) and 10 Black non-Hispanic (BNH) patients	NA	Breast microbiota by Illumina Sequencing	A total of 20 bacterial phyla and 419 genera were found in which Proteobacteria (59.4%) was the most common phylum, followed by Actinobacteria (19.1%), Firmicutes (17.7%), and Bacteroidetes (1.9%). Top 5 bacterial genera are *Ralstonia* (19.1%), *Staphylococcus* (6.4%), unclassified Bradyrhizobiaceae (5.5%), *Rubrobacter* (5.4%), and *Pseudomonas* (4.1%). Microbiota compositional differences were found between WNH and BNH groups of TNBC patients, but no significant differences were detected for TPBC patients. The β-diversity indexes of both WNH (AMOVA, *p* = 0.02) and BNH (AMOVA, *p* = 0.07) showed differences in tumour and normal tissue of TNBC patients. BNH patients with TNBC: Shannon diversity (*p* = 0.05) and evenness (*p* = 0.04) in tumour tissues were significantly lower than the normal adjacent tissue. WNH patients with TNBC: Shannon diversity (*p* = 0.04) and richness (ACE, *p* = 0.004; Chao1, *p* = 0.006) of tumour tissue were significantly higher than the normal adjacent tissue, and higher richness (ACE, *p* = 0.06; Chao1, *p* = 0.06) in tumour than in normal tissue. All with TPBC: No significant difference of microbial α-diversity between tumour and adjacent tissue, but higher richness (ACE, *p* = 0.04; Chao1, *p* = 0.05) of tumour tissue than the normal tissue was found.	(60)

NA, not available; BC, breast cancer; TPBC, triple-positive breast carcinoma; TNBC, triple-negative breast carcinoma; TILs, tumour−infiltrating lymphocytes; OTUs, operational taxonomic units; ACE, abundance-based coverage estimator; AMOVA, analysis of molecular variance.

**Table 4:** Characteristics of non-randomized intervention trials and their microbial profiling and diversity.

Author/year	Study period	Sample	Age (Mean ± *SD*)	Treatment	Microbial detection method	Bacterial profile and diversity	Ref.
Napeñas (2010)	2004-2006	Buccal mucosa samples from 9 newly diagnosed BC patients collected before and after chemotherapy (CTx)	53.3 ± 12.1	First-round CTx with adriamycin 60 mg/m^2^ and Cytoxan 600 mg/m^2^	Oral microbiota by ABI Sequencing	There were 41 species found in pre- (> 85%) and post-CTx samples, with *Gemella haemolysans* and *Streptococcus mitis* dominating. Seven species (17%) emerged only before treatment, while 25 (61%) appeared only after CTx. Species that appeared exclusively after CTx belong to Lachnospiraccae, *Acidaminococcus*, *Clostridiales*, *Oribacterium*, *Johnsonella*, *Peptostreptococcus*, *Aggregatibacter*, *Haemophilus*, Bacteroidetes, and species such as *Filifactor alocis*, *Veillonella parvula*, *Lactobacillus gasseri*, *Granulicatella adicans*, and *Selenomonas noxia*.	(63)
Frugé (2020)	2014-2017	Faecal samples from overweight and obese 32 EOBC patients were collected at baseline visits shortly after diagnosis and follow-up visits before surgery	61 ± 9	Attention-control arms: diet + exercise (average 30 ± 9 days) Weight-loss arm: diet + exercise + proper guidance (average 30 ± 9 days)	Gut microbiota by Illumina Sequencing	No significant relative abundance of *Akkermansia muciniphila* (AM) over time (*p* = 0.419) between low AM (LAM) and high AM (HAM). An additional 40 OTUs differed between LAM and HAM (*p* < 0.2), with more *Prevotella* and *Lactobacillus* genera in HAM *vs.* LAM and lower *Clostridium*, *Campylobacter*, and *Helicobacter*. Significant differences of β-diversity between LAM and HAM. (*p* = 0.002) Microbial richness and α-diversity were found to be larger in HAM (*p* < 0.05), with HAM individuals having roughly 25% more species present in stool samples at baseline. (*p* = 0.008)	(64)
Wu (2020)	NA	Faecal samples from 4 BC patients with no CTx, 13 with neo-adjuvant therapy, and 16 with adjuvant therapy.	50.6 ± 12.3	Neoadjuvant and adjuvant CTx, radiation, and surgery	Gut microbiota by Illumina Sequencing	In total, 13 taxa differed between those with HER2+ *vs.* HER2− tumours (*p* ≤ 0.001), 3 taxa between ER+ and ER− tumours, and 2 taxa between PR+ and PR− tumours. No significant α-diversity or phyla composition by ER/PR status, tumour grade, stage, parity, and body mass index, but had significant relationships with HER2 status and age at menarche. HER2+ *vs.* HER2− BC showed 12–23% lower α-diversity (*p* = 0.034), revealing low Firmicutes (*p* = 0.005), and high Bacteroidetes (*p* = 0.089).	(66)
Chiba (2020)	2004-2014	Fresh frozen tissue samples from 18 pre-treatment groups, 15 neoadjuvant CTx (Neo-CTx) groups, and 9 recurrence group	Pre-Tx: 65.3 ± 8.9 Neo-CTx: 58.9 ± 10.1 Recurrence: 64.3 ± 7.9	Neo-CTx (Doxorubicin Treatment)	Breast microbiota by Illumina Sequencing and breast tumour microarrays	Neo-CTx shifted the breast tumour microbiota, and specific microbes were correlated with tumour recurrence. No significant difference in bacterial load at phylum-level, but indicated a significant increase of *Pseudomonas* and drop of *Prevotella.* (*p* < 0.05) A significant reduction of bacterial diversity (*p* < 0.05) was found within the tumour. No alteration of bacterial load and diversity in the recurrence group, but provided a significant increase (*p* < 0.05) of *Brevundimonas* and *Staphylococcus* with no changes of *Pseudomonas* and *Prevotella.*	(65)
Guan (2020)	NA	Fresh frozen tissue samples from 15 BC patients and 16 benign patients	50	CTx with single-agent capecitabine in either conventional regimens (1,000–1,250 mg/m^2^ twice daily, given on days 1–14 every 3 weeks) or metronomic regimens (500 mg, thrice daily)	Breast microbiota by Illumina Sequencing	Lower unweighted-Unifrac index was found in the metronomic group than routine group. (*p* = 0.025) No significant drop in three α-diversity indices in the metronomic in compared with routine group. Patients with *Slackia* gut microbiota had a significantly shorter median progression-free survival (PFS) (9.2 *vs.* 32.7 months, *p* = 0.004) while patients with *Blautia obeum* had a longer PFS (32.7 *vs.* 12.9 months, *p* = 0.013).	(67)

NA, not available; BC, breast cancer; EOBC, early stage breast cancer; CTx, chemotherapy; Neo-CTx, neoadjuvant chemotherapy; OTUs, operational taxonomic units; ACE, abundance-based coverage estimator; AMOVA, analysis of molecular variance.

### 3.6 Microbial composition and diversity in breast cancer

**Figure 5:**
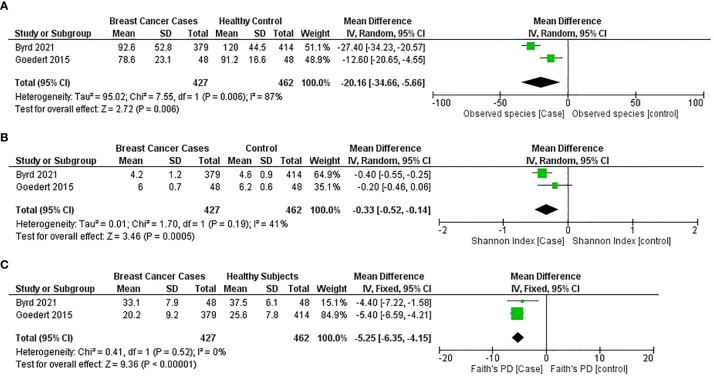
Meta-analysis forest plot representing the risk of breast cancer by different microbial profiling indexes like: **(A)** observed species; **(B)** Shannon index; and **(C)** faith’s PD.

A general significant reduction in gut bacterial species observed for the breast cancer group was found in two studies (44, 54) (*MD* = −20.16; 95% CI = −34.66 to −5.66; *p* = 0.006); however, the heterogeneity value is high (*I^2^* = 87%; *p* = 0.006) (**Figure 5**).

The general estimates of the Shannon index (*MD* = −0.35; 95% CI = −0.48 to −0.22; *p* < 0.00001) and the Faith PD index (*MD* = −5.25; 95% CI = −6.35 to −4.15; *p* < 0.001) from the studies (44, 54) reported that a significant reduction in gut microbial α-diversity was found in patients with breast cancer compared with healthy subjects (**Figures 5B, C**).

## 4 Discussion

The systematic review of the literature spanned three decades and included data from nearly a dozen countries on the breast, oral, or gut microbiome and breast cancer. Meta-analysis revealed microbial changes and diversity in breast cancer patients *versus* controls. The review bridged a gap that allowed us to connect previous microbiome studies in breast cancer patients using qualitative and quantitative meta-analysis tools.

Forest plots from two studies (44, 54) indicated that breast cancer patients have a lower α-diversity as measured by the Shannon index, observed species, and Faith’s phylogenetic diversity (PD), than healthy individuals. For the detection of microbial α-diversity, common parameters include Chao1, Fisher’s alpha, Faith’s PD, Simpson, Abundance-based coverage estimators (ACEs), and Good’s coverage indices (68). Some parameters simply count the number of species or operational taxonomic units (OTUs) present in an area, while others consider the abundance or frequency of the OTUs. As a result, most of the researchers used more than one diversity index, and combining and analysing different indexes in the current study were difficult; therefore, the number of studies for meta-analysis was limited.

The human intestinal microbiota is dominated by two bacterial phyla, Firmicutes and Bacteroidetes, which represent more than 90% of the total community, as well as other subdominant phyla such as Proteobacteria, Actinobacteria, and Verrucomicrobia (69). Bacteroidetes, which make up 45–55% of all bacteria, are all Gramme-negative bacteria (70). When they are particularly high, with values greater than 70–75%, it appears that they put the host at risk of diabetes and possibly other inflammatory diseases, especially when there is a high percentage of Proteobacteria present. Firmicutes, even if present in a lower percentage than Bacteroidetes, are believed to account for 40–45% of the total fecal microbiota under normal conditions (70). The study by Ma et al. found a reduced relative abundance of Firmicutes and Bacteroidetes, increased levels of Proteobacteria, Actinobacteria, and Verrucomicrobia at the phylum level, and decreased abundance of *Faecalibacterium prausnitzii* in fecal samples from 25 breast cancer patients (50). Furthermore, the Firmicutes/Bacteroidetes (F/B) ratio in breast cancer patients was significantly higher than in controls (53). It was also discovered that the absolute numbers of total bacteria and three bacterial groups (Firmicutes, *Faecalibacterium prausnitzii*, and *Blautia*) differed significantly according to the patient’s BMI, as shown in **Table 3** (55). All of these demonstrated the gut microbial pattern, as well as a transform in the F/B ratio, which contributes to an increased risk of breast cancer.

A study described that the breast microbiota of tumour tissue has Proteobacteria (48%), Actinobacteria (26.3%), Firmicutes (16.2%), and others (9.5%) of miscellaneous phyla (18). From the included studies (17, 43, 47, 48, 51, 56, 57, 60), an increased relative abundance of *Bacillus*, *Enterobacteriaceae*, *Staphylococcus*, *Fusobacterium*, *Atopobium*, *Gluconacetobacter, Hydrogenophaga*, *Lactobacillus*, *Corynebacterium*, *Actinomyces*, *Propionibacteriaceae*, *Clostridia*, *Bacteroidia*, *WPS_2*, *Ruminococcaceae*, *Acidaminococcus*, *Acinetobacter*, *Akkermansia*, *Bacteroides*, *Sutterella*, *Agrococcus*, *Ralstonia*, *Methylobacterium*, *and Sphingomonas* were also discovered. Furthermore, a distinct breast tissue microbiota was found in different breast skin tissues, breast skin swabs, buccal swabs, and deep microbial communities between benign and malignant breast disease (48). Furthermore, there appears to be a geographical difference between the Canadian and Irish breast tissue microbiome (**Figure 4**), but there is still evidence to prove that (48). Furthermore, the study showed a distinct profile of the breast tissue microbiome with increased species richness compared with the overlying skin tissue, suggesting that the differences may be due to the difference in their environment and ecosystem (48).

The researchers discovered a 10-fold increase in bacterial load in breast tumours, as well as an inverse correlation between bacterial count in tumour tissue and breast cancer stage, with stage 3 patients having the lowest 16*S* ribosomal DNA copy numbers in a study comparing the breast microbiota between breast tissue and their paired normal tissue (43). A significant increase in *Methylobacterium radiotolerans* (*p* = 0.015) was found in breast tumours, while *Sphingomonas yanoikuyae* (*p* = 0.009) was found in low abundance in paired normal tissue (43). However, a study found similarities in the breast microbiota between the tumour and adjacent normal tissues using weighted UniFrac distances (57). The number of breast microbiota was found to increase in breast tumours, but there was less diversity compared with normal tissues paired.

Identifying the microbiome of the four BC subtypes may reveal a link between the microbiota and the therapeutic response (71, 72). The oncobiome of each BC cancer subtype is unique and contains a wide range of microbial signatures. ER had the most diverse oncobiome, while TN had the least (31). Furthermore, the presence or absence of specific microbes distinguishes each BC subtype and, therefore, the level of detection of these microbes was predictive of patient outcomes.

Among the multiple drivers of microbial differences, a common element is menopausal status. We found that patients with premenopausal breast cancer had an increased fecal profile of *Enterobacteriaceae*, aerobic *Streptococci*, *Lactobacilli*, and anaerobic bacteria, including *Clostridia*, *Bacteroides*, and *Lactobacilli* (41). A similar behaviour of the anaerobic flora was found in patients with late menopause. The urine microbiome of peri/postmenopausal patients also showed a reduced abundance of *Lactobacilli* and an elevated profile of many genera, including but not limited to anaerobic bacteria such as *Varibaculum*, *Porphyromonas*, *Prevotella*, *Bacteroides*, and members of the Clostridia class (17). More details of the microbiota in different groups are described in **Tables 3**, **4**.

Although the role of species in the equilibrium of the GI environment is unclear, an increase in bacterial concentration can modulate estrogen metabolism through deconjugation and contribute to total bacterial enzyme activity (41). For example, the enzymes β-glucuronidase and β-glucosidase are produced by *E. coli* and *S. faecalis*, respectively. From an observation study of the enzymatic activity of fecal bacteria, it was recognised that the activity in postmenopausal women was lower than that of premenopausal cases. The expression of high to low enzymes, such as esterase C4, esterase-lipase, leucine and valine acrylamidase, acid phosphatase, α-galactosidase, and β-glucuronidase, was found in healthy subjects, while leucine and valine acrylamidase, β-glucuronidase, and esterase-lipase were higher in women with postmenopausal breast cancer (42). Therefore, it showed intersubject variability for enzymatic activities.

To explore the impact of cancer chemotherapy, Napenas et al. performed a profile of the oral microbiome on nine newly diagnosed breast cancer patients before and after receiving treatment (63). In general, 41 species were detected in total (**Supplementary Table S2**), and interestingly, > 85% of the detection (33/41) were newly identified species in chemotherapy patients. It revealed that seven species and 25 species appeared only before and after cancer chemotherapy, respectively, and the increase in species per patient had a mean of 2.6 (*SD* = 4.7, *p* = 0.052) after chemotherapy (63).

Chiba et al. (65) evaluated modulation of the tumour microbiome by neoadjuvant chemotherapy using breast tumour microarrays (**Table 5**). It demonstrated no significant changes in total bacterial load in untreated and treated patients; however, bacterial diversity was significantly reduced in the treated tumour. The classification at the phylum level did not show significant changes between the two groups, but the analysis at the genus level showed a significant elevation in *Pseudomonas* species and a reduction in the abundance of *Prevotella* in the treated cases (65). In addition, it indicated the modulation of chemotherapy in the tumour microbiome and the correlation of some genera in patients with tumour recurrence (65). Another study by Guan et al. showed significant differences in beta diversity before and after chemotherapy with single agent capecitabine and metronomic regimens (**Table 5**) and supported the reduction of bacterial diversity in the intervention group but was not statistically significant (67). In general, it was indicated that particular microorganisms are associated with tumour recurrence and that chemotherapy and neoadjuvant chemotherapy change the microbial composition and diversity in breast and oral tumours.

A study investigated the microbiome profile in fecal DNA only in women and control subjects; then, the case and control study discovered a significantly altered microbial community in cases (*p* = 0.006) compared with controls and less alpha diversity (*p* = 0.004) (17). Another study (66) revealed that early and late menarche was associated with a low number of OTU (*p* = 0.036), particularly reduced Firmicute expression (*p* = 0.048), and a low chao1 index (*p* = 0.020) (**Table 2**). Therefore, menopause and menarche status are associated with lower gut microbiome diversity, according to research, but more research is needed in large study populations to identify replicable patterns in taxa impacted by menopause (73, 74).

Goedert et al. (44) showed that a twofold higher level of estrogen expression was found in postmenopausal patients; however, the difference did not change the microbiota and the association with cancer. Banerjee et al. (2018) found that an increased abundance of *Brevundimonas* was detected in cases of ER+ breast cancer and triple-positive breast cancer (TPBC) compared with cases of ER^-^ breast cancer and TNBC. In addition, a high abundance of *Mobiluncus* and *Mycobacterium* was predominantly identified in ER breast cancer samples. Furthermore, *Acinetobacter* was the most prominent in HR^+^ breast cancer and HER2+ breast cancer cases, *Brevundimonas* in TPBC samples, and *Caulobacter* in TNBC samples (14). Interestingly, a distinct pattern of microbial profile was explored in patients with TNBC using pan-pathogen array technology and was summarised as in (**Table 5**) (46).

**Table 5:** Characteristics of case-control studies and their microbial profiling and diversity.

Author/year	Study period	Sample	Age (Mean ± *SD*)	Microbial detection method	Bacterial profile and diversity	Ref.
Minelli (1990)	NA	Faeces samples of 18 BC patients (4 PrM and 14 PoM) and 30 healthy subjects	NA	Gut microbiota by Microbial cultures	PrM patients: ↑ *Enterobacteriaceae* (*E. coli* and lactose non-fermenters; *p* < 0.001), aerobic *streptococci*, and *lactobacilli*; anaerobes (*Clostridia*, *Bacteroides*, and *Lactobacilli*; *p* < 0.001); No significant increase for anaerobic cocci. PoM < 5 years: ↑ *Bacteroides*, and *Clostridia*. (*p* < 0.001) PoM > 5 years: ↑ *Lactobacilli*, *E. coli*, *Enterobacteria*, *Bacteroides*, and *Clostridia*. (*p* < 0.05)	(41)
Benini (1992)	NA	Faeces samples of 28 BC patients and 45 healthy subjects	NA	Gut microbiota by Microbial cultures	The bacterial load and species of BC was significantly higher than that of healthy women. PrM patients: ↑ *E. coli*, aerobic and anaerobic *Lactobacilli*, *Bacteroides*, and *Clostridia.* (*p* < 0.01) PoM < 5 years: ↑ *E. coli* and *Bacteroides*, *↓ Enterococci.* (*p* < 0.01) PoM > 5 years: ↑ *E. coli*, *Bacteroides*, and *Clostridia* (*p* < 0.05); ↓ Fungi. (*p* < 0.01)	(42)
Goedert (2015)	NA	Faeces samples of 48 BC patients and 48 controls	62	Gut microbiota by Illumina Sequencing	Sequencing revealed 1,561 microbial taxa. A significant difference in genus composition of gut microbiota between patients and controls (unweighted UniFrac *p* = 0.009). Decreased α-diversity of gut microbiota (*p* ≤ 0.004) at patients than that of controls, except for the Shannon index (*p* = 0.09). Showed alteration of the composition of their IgA-positive (*p* = 0.02) and IgA-negative (*p* = 0.05) intestinal microbiota and significant reduction of α-diversity (*p* ≤ 0.05) in BC patients after adjusting for estrogens and other factors.	(44)
Goedert (2018)						(45)
Zhu (2018)	NA	Faeces samples of 62 BC patients and 71 controls	PrM: 37.06 *vs.* 35.52 PoM: 57.45 *vs.* 56.89	Gut microbiota by Illumina Sequencing	There were significant differences in the relative abundance of 45 species between PoM patients and controls; 38 species were enriched in PoM patients, including *E. coli, Klebsiella sp*_1_1_55, *Prevotella amnii, Enterococcus gallinarum, Actinomyces* sp. *HPA0247*, *Shewanella putrefaciens*, *Erwinia amylovora*, and 7 species were less abundant, including *Eubacterium eligens* and *L. vaginalis*.	(15)
Ma (2020)	2017-2018	Faeces samples of 25 BC patients and 25 benign patients as controls	NA	Gut microbiota by Illumina Sequencing	Found 49 significantly different flora and 26 different metabolites between groups. BC: ↓ Firmicutes, Bacteroidetes, and *Faecalibacterium;* ↑ Verrucomicrobiota, Proteobacteria, and Actinobacteria. Revealed significant difference of β-diversity between groups and lower α-diversity in BC group. (*p* < 0.05)	(50)
Uzan-Yulzari (2020)	NA	Faeces samples of 28 BC and 5 benign cancer patients	NA	Gut microbiota by Illumina Sequencing	It was divided into weight-gain (WG) and weight-loss (WL) groups based on the weight difference ≥ 3% after neoadjuvant chemotherapy. Higher relative abundance of members of the family Erysipelotrichaceae, the class Erysipelotrichia, and the order Erysipelotrichales was found in the WG group. Their β-diversity (*p* = 0.012) revealed significant differences between the groups, and significant increase in α-diversity (*p* = 0.01) in the WG women.	(52)
He (2021)	2019	Faeces samples of 54 BC patients and 28 controls	39.74 *vs.* 37.54	Gut microbiota by Illumina Sequencing	Firmicutes/Bacteroidetes ratio was largely higher in BC than controls. BC patients: ↓ Acidobacteria, Nitrospirae, Fusobacteria, and Cyanobacteria/Chloroplast; *↑* Synergistetes*;* the top 10 bacterial species that significantly decreased are *Allisonella, Megasphaera, Pediococcus, Abiotrophia, Granulicatella*, and *Clostridium_sensu_stricto* belonging to Firmicutes, *Serratia and Enhydrobacter* belonging to Proteobacteria, *Fusobacterium* belonging to Fusobacteria, and *Slackia* belonging to Actinobacteria. PrM BC patients and normal PrM women could be distinguished by *Pediococcus* and *Desulfovibrio*.	(53)
Byrd (2021)	NA	Faeces samples of 379 BC, 102 benign patients (NM), and 414 healthy controls (NC).	BC: 50.8 NM: 38.8 NC: 46.9	Gut microbiota by Illumina Sequencing	The α-diversity metrics are strongly and inversely associated with the odds of BC, and for those in the highest *vs.* lowest tertile of observed ASVs, the odds ratio was 0.21 (p_trend_ < 0.001). No significant difference of α-diversity for NM and BC grade/molecular subtype. The β-diversity distance matrices and multiple taxa with possible estrogen-conjugating and immune-related functions are associated with BC. (*p* < 0.001)	(54)
Xuan (2014)	NA	20 tumour tissue and 20 paired normal tissue	63.43	Breast microbiota by Pyrosequencing, Illumina	96.6% of all samples are Proteobacteria, Firmicutes, Actinobacteria, Bacteroidetes, and Verrucomicrobia. An inverse association of BC stage and bacterial load was observed in tumour tissue but not in paired normal tissue. BC patients (100%, *p* = 0.015): ↑ *Methylobacterium radiotolerans* (66.67%, 2/3 OTUs) Paired normal tissue (95%, *p* = 0.0097): ↑ *Sphingomonas yanoikuyae* (50%, 4/8 OTUs)	(43)
Urbaniak (2014)	2012	Breast tissue from Canadians including 27 BC, 11 benign, and 5 healthy subjects, and Irish accounting for 33 BC patients and 5 healthy	NA	Breast microbiota by Ion-Torrent sequencing	Obtained 121 OTUs based on 97% sequence similarity which includes 7 different phyla, 57 genera, and 25 species. Phyla: Proteobacteria, Firmicutes, Actinobacteria, Bacteroidetes, Deinococcus-Thermus, Verrucomicrobia, and Fusobacteria. Canadian: *Bacillus* (11.4%), *Acinetobacter* (10.0%), *Enterobacteriaceae* (8.3%), *Pseudomonas* (6.5%), *Staphylococcus* (6.5%), *Propionibacterium* (5.8%), *Comamonadaceae* (5.7%), *Gammaproteobacteria* (5.0%), and *Prevotella* (5.0%) Irish: *Enterobacteriaceae* (30.8%), *Staphylococcus* (12.7%), *Listeria welshimeri* (12.1%), *Propionibacterium* (10.1%), and *Pseudomonas* (5.3%)	(13)
Banerjee (2015)	NA	FFPE tissue samples from 100 BC patients, 17 adjacent healthy tissue of the patients, and 20 healthy subjects	NA	Breast microbiota by Illumina Sequencing and pan-pathogen Microarray	Prevalent viral, bacterial, fungal, and parasitic genomic sequences were detected in the TNBC samples. Bacterial profile in TNBC: Prevalence of *Arcanobacterium* (75%), followed by *Brevundimonas*, *Sphingobacteria, Providencia*, *Prevotella*, *Brucella*, *Eschherichia*, *Actinomyces*, *Mobiluncus*, *Propiniobacteria*, *Geobacillus*, *Rothia*, *Peptinophilus*, and *Capnocytophaga*.	(46)
Urbaniak (2016)	NA	Fresh frozen tissue of 45 BCs, 13 benign patients, and 23 healthy controls	53.5	Breast microbiota by Illumina Sequencing	The microbial profiles differed between normal adjacent tissue of BC patients and tissue from healthy controls (*p* < 0.01), and the similarity was found between the normal adjacent tissue and the tumour tissues. BC patients: ↑ *Bacillus*, *Staphylococcus*, Enterobacteriaceae, Comamondaceae, and Bacteroidetes. Healthy subjects: ↑ *Prevotella, Lactococcus*, *Streptococcus*, *Corynebacterium*, and *Micrococcus.*	(47)
Hieken (2016)	NA	Fresh frozen tissue of 17 BC and 16 benign patients	60	Breast microbiota by Illumina Sequencing	The microbiota of breast tissue, breast skin swabs, and buccal swabs differed from the microbiota of breast skin tissue, and also between BC and benign breast tissue. BC patients: ↑ *Fusobacterium*, *Atopobium, Gluconacetobacter, Hydrogenophaga*, and *Lactobacillus*	(48)
Wang (2017)	2014-2016	Breast tissue, mid-stream urine, and oral rinse from 57 BC patients and 21 healthy subjects	52.82	Breast, urine, and oral microbiota by Illumina Sequencing	Breast Microbiota No significant differences in α-diversity or microbial composition between BC and paired normal tissue in those patients. (p = 0.32) Significant difference of breast microbiota between groups (p = 0.03) at which decreased Methylobacterium (p = 0.03) were found. Oral Microbiota No significant compositional differences and diversities of oral microbiota. Urine Microbiota No distinct clusters between groups (*p* = 0.09). It was largely different by menopausal status (*p* = 0.02), with peri/post-menopausal women showing reduced *Lactobacillus.* Independent of menopausal status, BC patients had high gram-positive organisms including *Corynebacterium* (*p* < 0.01), *Staphylococcus* (*p* = 0.02), *Actinomyces* (*p* < 0.01), and Propionibacteriaceae (*p* < 0.01).	(17)
Thompson (2017)	NA	Frozen tissue blocks of 668 BC and 72 healthy subjects.	NA	Breast microbiota by Illumina Sequencing	BC tissue: Proteobacteria (48%), Actinobacteria (26.3%), Firmicutes (16.2%), Others (9.5%) from various phyla; ↑ abundance for *S. pyogenes* and *L. rossiae. L. fleischmannii* and, to a lesser extent, *N. Subflava* were found. *H. influenza* was associated with genes involved in tumour growth pathways. Non-cancerous adjacent tissue: The abundance ranges from 0.5 to 19.3%, though these species make up 85.64% of the entire microbiota in breast tissue.	(18)
Banerjee (2018)	NA	FFPE tissue samples from 148 BC patients and 20 healthy subjects.	NA	Breast microbiota by Illumina Sequencing and Microarray	The TNBC and TPBC samples had unique patterns, however, the ER+ and HER2+ samples had comparable microbial signatures. Fungi: only 7 fungal families (*Aspergillus, Candida, Coccidioides, Cunninghamella, Geotrichum, Pleistophora*, and *Rhodotorula*). Parasites: *Ancylostoma*, *Angiostrongylus*, *Echinococcus*, *Sarcocystis*, *Trichomonas*, *and Trichostrongylus* were uniquely associated with TPBC. *Balamuthia* signatures were associated significantly with hormonal BC samples, and that of *Centrocestus*, *Contracaecum*, *Leishmania*, *Necator*, *Onchocerca*, *Toxocara*, *Trichinella*, and *Trichuris* were detected significantly only with TNBC samples.	(14)
Smith (2019)	NA	Fresh frozen tissue from 64 BC patients, 11 adjacent healthy tissue of the patients, and 8 healthy subjects.	45	Breast microbiota by Illumina Sequencing	Normal *vs.* Tumour: ↓ Pseudomonadaceae (Proteobacteria), Sphingomonadaceae (Bacteroidetes), and Ruminococcaceae (Firmicutes); ↑ Actinomycetaceae (Actinobacteria) Tumour: ↑*Clostridia*, *Bacteroidia*, *WPS_2*, Ruminococcaceae (LDA > 4) Normal pairs: ↑ Pseudomonadaceae, Sphingomonadaceae, and Caulobacteraceae (LDA > 5). Stage 1: ↑ Proteobacteria; Ruminococcaceae (Firmicutes), and *Hyphomicrobium* (Proteobacteria). Stage 2: ↑ Euryarchaeota, Firmicutes, and Spirochaetes; *Sporosarcina* (Firmicutes) Stage 3: ↑ *Thermi*, Gemmatimonadetes, and Tenericutes; *Bosea* (Proteobacteria) Luminal A tumours: ↑ Xanthomonadales (Proteobacteria) (LDA > 5); Luminal B tumours: ↑ *Clostridium* (Firmicutes); HER2 tumours: ↑ *Akkermasia* (Verrucomicrobia) (LDA = 4) TNBC: Streptococcaceae in TNBC; also Streptococcaceae, *Ruminococcus* (both Firmicutes) (LDA > 3.5)	(49)
Klann (2020)	NA	Breast tissue samples of 10 BC patients and 36 healthy samples.	NA	Breast microbiota by Illumina Sequencing	There were significant differences in bacterial diversity between tumour and normal breast tissue, as well as differences in composition between women and breasts from the same woman. The most abundant phyla are Bacteroidetes, Firmicutes, Proteobacteria, and Actinobacter. The most abundant OTUs are Ruminococcaceae and *Acidaminococcus*, *Acinetobacter*, *Akkermansia*, *Bacteroides*, and *Sutterella*.	(51)
Huang (2018)	2006-2015	cfDNA of 3 BC patients and 2 healthy subjects	NA	Breast microbiota by Illumina Sequencing	Bacterial species were more diverse and more likely to be present at high levels in EOBC patients. *Acinetobacter johnsonii XBB1* and low *Mycobacterium* spp. were discovered in all healthy females but were also found in an EOBC patient, but large titers of bacterial cfDNA in EOBC patients were obtained from *Pseudomonas* or *Sphingomonas* spp.	(19)

NA, not available; BC, breast cancer; IgA, immunoglobulin-A; PrM, pre-menopausal patients; PoM, post-menopausal patients; EOBC, early breast cancer; OTUs, operational taxonomic units; cfDNA, cell-free DNA.

A study also explored the level of expression of antibacterial response genes in tumour tissue, paired normal tissue, and healthy tissue and found that a third of antibacterial genes were significantly down-regulated in breast tumour cases after normalising with a housekeeping gene β-actin, interestingly there are no more up-regulated genes (43). Furthermore, a significant reduction was observed in the transcripts of the microbial sensors, Toll-like receptors (TLR)–2, TLR-5, and TLR-9 (*p* = 0.0298, *p* = 0.0201, and *p* = 0.0021, respectively) was observed in tumour tissue while there was a similar expression level of TLR1, TLR4, and TLR6 in healthy and tumour tissue. Furthermore, tumour tissues showed significantly decreased expression of cytoplasmic microbial sensors (NOD1 and NOD2) and downstream signaling molecules for innate microbial sensors such as CARD6, CARD9, and TRAF6 (*p* = 0.0207, *p* = 0.0040, and *p* = 0.0119, respectively). The levels of bactericidal/permeability increasing protein (BPI), myeloperoxidase (MPO) and proteinase 3 (PRTN3) are significantly reduced (*p* = 0.0133, 0.002, and 0.0022, respectively) (46). Although further research is needed to confirm the influence of the local microenvironment of breast tissue, these findings demonstrated a significant decrease in antimicrobial responses in breast tumour tissue (43).

However, the meta-analysis study has some limitations. First, several matrices for detecting alpha diversity were utilised in different studies, and there are probably no standardised tools for measurement. Therefore, only a few studies were able to analyse quantitatively. Second, some mean values cannot be found in the papers and supplementary files, and the email contacts for 2 weeks were reachable only to some; perhaps the contacts were changed, or the data were not archived for a long period. Therefore, qualitative analysis was applied to the data provided for the articles.

In general, our meta-analysis suggests the fecal, tumour, or oral microbiome profile of breast cancer patients, differences in microbiota abundance by menopausal status, menarche and cancer stages, and the change in the microbial pattern before and after chemotherapy. However, the microbiome investigation is still in its infancy for breast cancer patients, and the sample size is normally limited due to high sequencing costs. Therefore, more studies with a larger cohort of patients would be required to identify the biological and pathological significance of the findings in the meta-analysis. We expected that the review could fill the gap linking to better understand the connection between breast cancer and the microbiome.

## Data availability statement

The original contributions presented in the study are included in the article/**Supplementary Material**. Further inquiries can be directed to the corresponding author.

## Author contributions

KP and MT contributed for the conceptualization of the study. MT, TN, and KP conducted the methodology. KP and MT applied the software for data visualization. MT, TN, and KC performed the investigation throughout the study. TN, MT, and KP made the formal analysis. MT, KC, TN completed the data curation. MT, and KP prepared the original draft writing. For review and editing, MT, and KP emphasized on it. NH, and KP involved for supervision. KP, and NH worked on the project administration. All authors contributed to the article and approved the submitted version.

## References

[1] Sung H Ferlay J Siegel RL Laversanne M Soerjomataram I Jemal A . Global cancer statistics 2020: GLOBOCAN estimates of incidence and mortality worldwide for 36 cancers in 185 countries. CA Cancer J Clin (2021) 7(3):209–49. doi: 10.3322/caac.21660 33538338

[2] Loibl S Poortmans P Morrow M Denkert C Curigliano G . Breast cancer. Lancet (2021) 397(10286):1750–69. doi: 10.1016/S0140-6736(20)32381-3 33812473

[3] Dashti SG Simpson JA Karahalios A Viallon V Moreno-Betancur M Gurrin LC . Adiposity and estrogen receptor-positive, postmenopausal breast cancer risk: Quantification of the mediating effects of fasting insulin and free estradiol. Int J Cancer (2020) 146(6):1541–52. doi: 10.1002/ijc.32504 31187481

[4] Nyante SJ Gammon MD Kaufman JS Bensen JT Lin DY Barnholtz-Sloan JS . Genetic variation in estrogen and progesterone pathway genes and breast cancer risk: an exploration of tumor subtype-specific effects. Cancer Causes Control (2015) 26(1):121–31. doi: 10.1007/s10552-014-0491-2 PMC4291841 25421376

[5] Bogdanova N Helbig S Dork T . Hereditary breast cancer: ever more pieces to the polygenic puzzle. Hered Cancer Clin Pract (2013) 11(1):12. doi: 10.1186/1897-4287-11-12 24025454 PMC3851033

[6] Flores R Shi J Fuhrman B Xu X Veenstra TD Gail MH . Fecal microbial determinants of fecal and systemic estrogens and estrogen metabolites: a cross-sectional study. J Transl Med (2012) 10:253. doi: 10.1186/1479-5876-10-253 23259758 PMC3552825

[7] Hussain T Murtaza G Kalhoro DH Kalhoro MS Metwally E Chughtai MI . Relationship between gut microbiota and host-metabolism: Emphasis on hormones related to reproductive function. Anim Nutr (2021) 7(1):1–10. doi: 10.1016/j.aninu.2020.11.005 33997325 PMC8110851

[8] Gilbert JA Blaser MJ Caporaso JG Jansson JK Lynch SV Knight R . Current understanding of the human microbiome. Nat Med (2018) 24(4):392–400. doi: 10.1038/nm.4517 29634682 PMC7043356

[9] Sepsis Lung Microbiome Study G . Could lung bacterial dysbiosis predict ICU mortality in patients with extra-pulmonary sepsis? A proof-of-concept study. Intensive Care Med (2020) 46(11):2118–20. doi: 10.1007/s00134-020-06190-4 32767076

[10] Zhuang H Cheng L Wang Y Zhang YK Zhao MF Liang GD . Dysbiosis of the gut microbiome in lung cancer. Front Cell Infect Microbiol (2019) 9:112. doi: 10.3389/fcimb.2019.00112 31065547 PMC6489541

[11] Sekyere JO Maningi NE Matukane SR Mbelle NM Fourie PB . An Oxford nanopore-based characterisation of sputum microbiota dysbiosis in patients with tuberculosis: from baseline to 7 days after antibiotic treatment. medRxiv (2021).

[12] Laborda-Illanes A Sanchez-Alcoholado L Dominguez-Recio ME Jimenez-Rodriguez B Lavado R Comino-Mendez I . Breast and gut microbiota action mechanisms in breast cancer pathogenesis and treatment. Cancers (Basel) (2020) 12(9):2465–92. doi: 10.3390/cancers12092465 PMC7565530 32878124

[13] Urbaniak C Cummins J Brackstone M Macklaim JM Gloor GB Baban CK . Microbiota of human breast tissue. Appl Environ Microbiol (2014) 80(10):3007–14. doi: 10.1128/AEM.00242-14 PMC4018903 24610844

[14] Banerjee S Tian T Wei Z Shih N Feldman MD Peck KN . Distinct microbial signatures associated with different breast cancer types. Front Microbiol (2018) 9:951. doi: 10.3389/fmicb.2018.00951 29867857 PMC5962706

[15] Zhu J Liao M Yao Z Liang W Li Q Liu J . Breast cancer in postmenopausal women is associated with an altered gut metagenome. Microbiome (2018) 6(1):136. doi: 10.1186/s40168-018-0515-3 30081953 PMC6080540

[16] Tzeng A Sangwan N Jia M Liu C-C Keslar KS Downs-Kelly E . Human breast microbiome correlates with prognostic features and immunological signatures in breast cancer. Genome Med (2021) 13(1):60. doi: 10.1186/s13073-021-00874-2 33863341 PMC8052771

[17] Wang H Altemus J Niazi F Green H Calhoun BC Sturgis C . Breast tissue, oral and urinary microbiomes in breast cancer. Oncotarget (2017) 8(50):88122–38. doi: 10.18632/oncotarget.21490 PMC5675698 29152146

[18] Thompson KJ Ingle JN Tang X Chia N Jeraldo PR Walther-Antonio MR . A comprehensive analysis of breast cancer microbiota and host gene expression. PloS One (2017) 12(11):e0188873. doi: 10.1371/journal.pone.0188873 29190829 PMC5708741

[19] Huang YF Chen YJ Fan TC Chang NC Midha MK Chen TH . Analysis of microbial sequences in plasma cell-free DNA for early-onset breast cancer patients and healthy females. BMC Med Genomics (2018) 11(Suppl 1):16. doi: 10.1186/s12920-018-0329-y 29504912 PMC5836824

[20] Kovacs T Miko E Vida A Sebo E Toth J Csonka T . Cadaverine, a metabolite of the microbiome, reduces breast cancer aggressiveness through trace amino acid receptors. Sci Rep (2019) 9(1):1300. doi: 10.1038/s41598-018-37664-7 30718646 PMC6361949

[21] Al-Ansari MM AlMalki RH Dahabiyeh LA Abdel Rahman AM . Metabolomics-microbiome crosstalk in the breast cancer microenvironment. Metabolites. (2021) 11(11):758–70. doi: 10.3390/metabo11110758 PMC8619468 34822416

[22] Miko E Kovacs T Sebo E Toth J Csonka T Ujlaki G . Microbiome-microbial metabolome-cancer cell interactions in breast cancer-familiar, but unexplored. Cells (2019) 8(4):293–326. doi: 10.3390/cells8040293 PMC6523810 30934972

[23] Sui Y Wu J Chen J . The role of gut microbial beta-glucuronidase in estrogen reactivation and breast cancer. Front Cell Dev Biol (2021) 9:631552. doi: 10.3389/fcell.2021.631552 34458248 PMC8388929

[24] Mirzaei R Afaghi A Babakhani S Sohrabi MR Hosseini-Fard SR Babolhavaeji K . Role of microbiota-derived short-chain fatty acids in cancer development and prevention. BioMed Pharmacother (2021) 139:111619. doi: 10.1016/j.biopha.2021.111619 33906079

[25] Režen T Rozman D Kovács T Kovács P Sipos A Bai P . The role of bile acids in carcinogenesis. Cell Mol Life Sci (2022) 79(5):243. doi: 10.1007/s00018-022-04278-2 35429253 PMC9013344

[26] Durack J Lynch SV . The gut microbiome: Relationships with disease and opportunities for therapy. J Exp Med (2019) 216(1):20–40. doi: 10.1084/jem.20180448 30322864 PMC6314516

[27] Rashidi A Ebadi M Rehman TU Elhusseini H Nalluri H Kaiser T . Gut microbiota response to antibiotics is personalized and depends on baseline microbiota. Microbiome. (2021) 9(1):211. doi: 10.1186/s40168-021-01170-2 34702350 PMC8549152

[28] Bose M Mukherjee P . Role of microbiome in modulating immune responses in cancer. Mediators Inflamm (2019) 2019:4107917. doi: 10.1155/2019/4107917 31308831 PMC6594313

[29] Terrisse S Derosa L Iebba V Ghiringhelli F Vaz-Luis I Kroemer G . Intestinal microbiota influences clinical outcome and side effects of early breast cancer treatment. Cell Death Differ (2021) 28(9):2778–96. doi: 10.1038/s41418-021-00784-1 PMC8408230 33963313

[30] Dieleman S Aarnoutse R Ziemons J Kooreman L Boleij A Smidt M . Exploring the potential of breast microbiota as biomarker for breast cancer and therapeutic response. Am J Pathol (2021) 191(6):968–82. doi: 10.1016/j.ajpath.2021.02.020 33713687

[31] Banerjee S Wei Z Tian T Bose D Shih NNC Feldman MD . Prognostic correlations with the microbiome of breast cancer subtypes. Cell Death Dis (2021) 12(9):831. doi: 10.1038/s41419-021-04092-x 34482363 PMC8418604

[32] Nougayrede JP Homburg S Taieb F Boury M Brzuszkiewicz E Gottschalk G . Escherichia coli induces DNA double-strand breaks in eukaryotic cells. Science (2006) 313(5788):848–51. doi: 10.1126/science.1127059 16902142

[33] Rubinstein MR Wang X Liu W Hao Y Cai G Han YW . Fusobacterium nucleatum promotes colorectal carcinogenesis by modulating e-cadherin/beta-catenin signaling *via* its FadA adhesin. Cell Host Microbe (2013) 14(2):195–206. doi: 10.1016/j.chom.2013.07.012 23954158 PMC3770529

[34] Wang TC Goldenring JR Dangler C Ito S Mueller A Jeon WK . Mice lacking secretory phospholipase A2 show altered apoptosis and differentiation with helicobacter felis infection. Gastroenterology (1998) 114(4):675–89. doi: 10.1016/S0016-5085(98)70581-5 9516388

[35] Page MJ Moher D Bossuyt PM Boutron I Hoffmann TC Mulrow CD . PRISMA 2020 explanation and elaboration: Updated guidance and exemplars for reporting systematic reviews. BMJ (2021) 372:n160. doi: 10.1136/bmj.n160 33781993 PMC8005925

[36] Huang X Lin J Demner-Fushman D . Evaluation of PICO as a knowledge representation for clinical questions. AMIA Annu Symp Proc (2006) 2016:359–63. PMC1839740 17238363

[37] Morgan RL Whaley P Thayer KA Schunemann HJ . Identifying the PECO: A framework for formulating good questions to explore the association of environmental and other exposures with health outcomes. Environ Int (2018) 121(Pt 1):1027–31. doi: 10.1016/j.envint.2018.07.015 PMC6908441 30166065

[38] Sterne JA Hernan MA Reeves BC Savovic J Berkman ND Viswanathan M . ROBINS-I: A tool for assessing risk of bias in non-randomised studies of interventions. BMJ (2016) 355:i4919. doi: 10.1136/bmj.i4919 27733354 PMC5062054

[39] Wells GA Shea B O'Connell D Peterson J Welch V Losos M . The Newcastle-Ottawa Scale (NOS) for assessing the quality of nonrandomised studies in meta-analyses. (2021). Available at: http://www.ohri.ca/programs/clinical_epidemiology/oxford.asp

[40] Higgins JPT Altman DG JACS . Chapter 8: Assessing risk of bias in included studies. In: Higgins JPT Churchill R Chandler J MS. C , editors. Cochrane handbook for systematic reviews of interventions. version 5.2.0. Cochrane (2017). Available at: https://training.cochrane.org/handbook.

[41] Bertazzoni Minelli E Beghini AM Vesentini S Marchiori L Nardo G Cerutti R . Intestinal microflora as an alternative metabolic source of estrogens in women with uterine leiomyoma and breast cancer. NY Acad Sci (1990). doi: 10.1111/j.1749-6632.1990.tb34337.x

[42] Benini A Bertazzoni EM Vesentini S Marchiori L Nardo G . Intestinal ecosystem and metabolic capacity in women with breast cancer. Pharmacol Res (1992) 25:184–5. doi: 10.1016/1043-6618(92)90352-C

[43] Xuan C Shamonki JM Chung A Dinome ML Chung M Sieling PA . Microbial dysbiosis is associated with human breast cancer. PloS One (2014) 9(1):e83744. doi: 10.1371/journal.pone.0083744 24421902 PMC3885448

[44] Goedert JJ Jones G Hua X Xu X Yu G Flores R . Investigation of the association between the fecal microbiota and breast cancer in postmenopausal women: a population-based case-control pilot study. J Natl Cancer Inst (2015) 107(8):184–5. doi: 10.1093/jnci/djv147 PMC4554191 26032724

[45] Goedert JJ Hua X Bielecka A Okayasu I Milne GL Jones GS . Postmenopausal breast cancer and oestrogen associations with the IgA-coated and IgA-noncoated faecal microbiota. Br J Cancer (2018) 118(4):471–9. doi: 10.1038/bjc.2017.435 PMC5830593 29360814

[46] Banerjee S Wei Z Tan F Peck KN Shih N Feldman M . Distinct microbiological signatures associated with triple negative breast cancer. Sci Rep (2015) 5:15162. doi: 10.1038/srep15162 26469225 PMC4606812

[47] Urbaniak C Gloor GB Brackstone M Scott L Tangney M Reid G . The microbiota of breast tissue and its association with breast cancer. Appl Environ Microbiol (2016) 82(16):5039–48. doi: 10.1128/AEM.01235-16 PMC4968547 27342554

[48] Hieken TJ Chen J Hoskin TL Walther-Antonio M Johnson S Ramaker S . The microbiome of aseptically collected human breast tissue in benign and malignant disease. Sci Rep (2016) 6:30751. doi: 10.1038/srep30751 27485780 PMC4971513

[49] Smith A Pierre JF Makowski L Tolley E Lyn-Cook B Lu L . Distinct microbial communities that differ by race, stage, or breast-tumor subtype in breast tissues of non-Hispanic black and non-Hispanic white women. Sci Rep (2019) 9(1):11940. doi: 10.1038/s41598-019-48348-1 31420578 PMC6697683

[50] Ma J Sun L Liu Y Ren H Shen Y Bi F . Alter between gut bacteria and blood metabolites and the anti-tumor effects of faecalibacterium prausnitzii in breast cancer. BMC Microbiol (2020) 20(1):82. doi: 10.1186/s12916-020-01751-2 32272885 PMC7144064

[51] Klann E Williamson JM Tagliamonte MS Ukhanova M Asirvatham JR Chim H . Microbiota composition in bilateral healthy breast tissue and breast tumors. Cancer Causes Control (2020) 31(11):1027–38. doi: 10.1007/s10552-020-01338-5 32844256

[52] Uzan-Yulzari A Morr M Tareef-Nabwani H Ziv O Magid-Neriya D Armoni R . The intestinal microbiome, weight, and metabolic changes in women treated by adjuvant chemotherapy for breast and gynecological malignancies. BMC Med (2020) 18(1):281. doi: 10.1186/s12916-020-01751-2 33081767 PMC7576808

[53] He C Liu Y Ye S Yin S Gu J . Changes of intestinal microflora of breast cancer in premenopausal women. Eur J Clin Microbiol Infect Dis (2021) 40(3):503–13. doi: 10.1007/s10096-020-04036-x 32936397

[54] Byrd DA Vogtmann E Wu Z Han Y Wan Y Clegg-Lamptey JN . Associations of fecal microbial profiles with breast cancer and nonmalignant breast disease in the Ghana breast health study. Int J Cancer (2021) 148(11):2712–23. doi: 10.1002/ijc.33473 PMC8386185 33460452

[55] Luu TH Michel C Bard JM Dravet F Nazih H Bobin-Dubigeon C . Intestinal proportion of blautia sp. is associated with clinical stage and histoprognostic grade in patients with early-stage breast cancer. Nutr Cancer (2017) 69(2):267–75. doi: 10.1080/01635581.2017.1263750 28094541

[56] Meng S Chen B Yang J Wang J Zhu D Meng Q . Study of microbiomes in aseptically collected samples of human breast tissue using needle biopsy and the potential role of *in situ* tissue microbiomes for promoting malignancy. Front Oncol (2018) 8:318. doi: 10.3389/fonc.2018.00318 30175072 PMC6107834

[57] Costantini L Magno S Albanese D Donati C Molinari R Filippone A . Characterization of human breast tissue microbiota from core needle biopsies through the analysis of multi hypervariable 16S-rRNA gene regions. Sci Rep (2018) 8(1):16893. doi: 10.1038/s41598-018-35329-z 30442969 PMC6237987

[58] Shi J Geng C Sang M Gao W Li S Yang S . Effect of gastrointestinal microbiome and its diversity on the expression of tumor-infiltrating lymphocytes in breast cancer. Oncol Lett (2019) 17(6):5050–6. doi: 10.3389/fonc.2018.00318 PMC6507298 31186716

[59] Yoon HJ Kim HN Bang JI Lim W Moon BI Paik NS . Physiologic intestinal (18)F-FDG uptake is associated with alteration of gut microbiota and proinflammatory cytokine levels in breast cancer. Sci Rep (2019) 9(1):18273. doi: 10.1038/s41598-019-54680-3 31797893 PMC6892830

[60] Thyagarajan S Zhang Y Thapa S Allen MS Phillips N Chaudhary P . Comparative analysis of racial differences in breast tumor microbiome. Sci Rep (2020) 10(1):14116. doi: 10.1038/s41598-020-71102-x 32839514 PMC7445256

[61] Di Modica M Gargari G Regondi V Bonizzi A Arioli S Belmonte B . Gut microbiota condition the therapeutic efficacy of trastuzumab in HER2-positive breast cancer. Cancer Res (2021) 81(8):2195–206. doi: 10.1158/0008-5472.CAN-20-1659 33483370

[62] Yao ZW Zhao BC Yang X Lei SH Jiang YM Liu KX . Relationships of sleep disturbance, intestinal microbiota, and postoperative pain in breast cancer patients: a prospective observational study. Sleep Breath (2021) 25(3):1655–64. doi: 10.1007/s11325-020-02246-3 PMC8376716 33211236

[63] Napenas JJ Brennan MT Coleman S Kent ML Noll J Frenette G . Molecular methodology to assess the impact of cancer chemotherapy on the oral bacterial flora: a pilot study. Oral Surg Oral Med Oral Pathol Oral Radiol Endod (2010) 109(4):554–60. doi: 10.1016/j.tripleo.2009.11.015 20303053

[64] Fruge AD van der Pol W Rogers LQ Morrow CD Tsuruta Y Demark-Wahnefried W . Fecal akkermansia muciniphila is associated with body composition and microbiota diversity in overweight and obese women with breast cancer participating in a presurgical weight loss trial. J Acad Nutr Diet (2020) 120(4):650–9. doi: 10.1016/j.jand.2018.08.164 PMC6509025 30420171

[65] Chiba A Bawaneh A Velazquez C Clear KYJ Wilson AS Howard-McNatt M . Neoadjuvant chemotherapy shifts breast tumor microbiota populations to regulate drug responsiveness and the development of metastasis. Mol Cancer Res (2020) 18(1):130–9. doi: 10.1158/1541-7786.MCR-19-0451 PMC9153322 31628201

[66] Wu AH Tseng C Vigen C Yu Y Cozen W Garcia AA . Gut microbiome associations with breast cancer risk factors and tumor characteristics: A pilot study. Breast Cancer Res Treat (2020) 182(2):451–63. doi: 10.1007/s10549-020-05702-6 PMC7297869 32468338

[67] Guan X Ma F Sun X Li C Li L Liang F . Gut microbiota profiling in patients with HER2-negative metastatic breast cancer receiving metronomic chemotherapy of capecitabine compared to those under conventional dosage. Front Oncol (2020) 10:902. doi: 10.3389/fonc.2020.00902 32733788 PMC7358584

[68] Thukral AK . A review on measurement of alpha diversity in biology. Agric Res J (2017) 54(1):10. doi: 10.5958/2395-146X.2017.00001.1

[69] Qin J Li R Raes J Arumugam M Burgdorf KS Manichanh C . A human gut microbial gene catalogue established by metagenomic sequencing. Nature (2010) 464(7285):59–65. doi: 10.1038/nature08821 20203603 PMC3779803

[70] Cazzaniga M Zonzini GB Di Pierro F Moricoli S Bertuccioli A . Gut microbiota, metabolic disorders and breast cancer: Could berberine turn out to be a transversal nutraceutical tool? A narrative analysis. Int J Mol Sci (2022) 23(20):12538. doi: 10.3390/ijms232012538 36293390 PMC9604377

[71] Rea D Coppola G Palma G Barbieri A Luciano A Del Prete P . Microbiota effects on cancer: from risks to therapies. Oncotarget (2018) 9(25):17915–27. doi: 10.18632/oncotarget.24681 PMC5915165 29707157

[72] Zitvogel L Ma Y Raoult D Kroemer G Gajewski TF . The microbiome in cancer immunotherapy: Diagnostic tools and therapeutic strategies. Science (2018) 359(6382):1366–70. doi: 10.1126/science.aar6918 29567708

[73] Peters BA Santoro N Kaplan RC Qi Q . Spotlight on the gut microbiome in menopause: Current insights. Int J Womens Health (2022) 14:1059–72. doi: 10.2147/IJWH.S340491 PMC9379122 35983178

[74] Calcaterra V Rossi V Massini G Regalbuto C Hruby C Panelli S . Precocious puberty and microbiota: The role of the sex hormone-gut microbiome axis. Front Endocrinol (Lausanne) (2022) 13:1000919. doi: 10.3389/fendo.2022.1000919 36339428 PMC9634744

