# Supplementary material for: Human gut, breast, and oral microbiome in breast cancer: A systematic review and meta-analysis
Source: Front Oncol. 2023 Mar 17;13:1144021. doi: 10.3389/fonc.2023.1144021 (PMC10063924; doi:10.3389/fonc.2023.1144021)
Supplement: Supplementary file 1 [file DataSheet_1.docx]

Supplementary Material

Human gut, breast and oral microbiome in breast cancer: A systematic review and meta-analysis

May Soe Thu^1,2,3^, Korn Chotirosniramit^4^, Tanawin Nopsopon^4,5,6^, Nattiya Hirankarn^2^, Krit Pongpirul^4,6,7,8^*

*** Correspondence:** Corresponding Author: doctorkrit@gmail.com

# Supplementary Tables

Supplementary table 1. Full search strategy.

| **Set #** | **PubMed** | **Results** |
| --- | --- | --- |
| 1  Microbiome | "microbiota"[MeSH Terms] OR "gastrointestinal microbiome"[MeSH Terms] OR "mycobiome"[MeSH Terms] OR microbiome*[tiab] OR microbiota*[tiab] OR microbial[tiab] OR microbe*[tiab] OR microflora*[tiab] OR flora*[tiab] OR microorganism*[tiab] OR pathobiont*[tiab] OR mycobiome*[tiab] OR mycobiota*[tiab] OR virome*[tiab] OR phylotype*[tiab] OR enterotype*[tiab] | **403932** |
| 2  Breast Cancer | "breast neoplasms"[MeSH Terms] OR (breast[tiab] AND (cancer*[tiab] OR neoplas*[tiab] OR tumor*[tiab] OR tumour*[tiab] OR malignan*[tiab] OR carcinoma*[tiab] OR adenocarcinoma*[tiab])) | **429035** |
| 3 | #1 AND #2 | **945** |
| 4 | animals[MeSH Terms] NOT humans[MeSH Terms] | **4805329** |
| 5 | #3 NOT #4 | **896** |
| **Set #** | **Embase** | **Results** |
| 1  Microbiome | 'microbiome'/exp OR 'microflora'/exp OR 'intestine flora'/exp OR 'mycobiome'/exp OR 'microbiome*':ti,ab OR 'microbiota*':ti,ab OR 'microbial':ti,ab OR 'microbe*':ti,ab OR 'microflora*':ti,ab OR 'flora*':ti,ab OR 'microorganism*':ti,ab OR 'pathobiont*':ti,ab OR 'mycobiome*':ti,ab OR 'mycobiota*':ti,ab OR 'virome*':ti,ab OR 'phylotype*':ti,ab OR 'enterotype*':ti,ab | **490885** |
| 2  Breast Cancer | 'breast cancer'/exp OR ('breast':ti,ab AND ('cancer*':ti,ab OR 'neoplas*':ti,ab OR 'tumor*':ti,ab OR 'tumour*':ti,ab OR 'malignan*':ti,ab OR 'carcinoma*':ti,ab OR 'adenocarcinoma*':ti,ab)) | **642120** |
| 3 | #1 AND #2 | **1947** |
| 4 | [animals]/lim NOT [humans]/lim | **5945936** |
| 5 | #3 NOT #4 | **1775** |
| **Set #** | **CENTRAL** | **Results** |
| 1  Microbiome | [mh microbiota] OR [mh "gastrointestinal microbiome"] OR [mh mycobiome] OR microbiome*:ti,ab,kw OR microbiota*:ti,ab,kw OR microbial:ti,ab,kw OR microbe*:ti,ab,kw OR microflora*:ti,ab,kw OR flora*:ti,ab,kw OR microorganism*:ti,ab,kw OR pathobiont*:ti,ab,kw OR mycobiome*:ti,ab,kw OR mycobiota*:ti,ab,kw OR virome*:ti,ab,kw OR phylotype*:ti,ab,kw OR enterotype*:ti,ab,kw | **17075** |
| 2  Breast Cancer | [mh "breast neoplasms"] OR (breast:ti,ab,kw AND (cancer*:ti,ab,kw OR neoplas*:ti,ab,kw OR tumor*:ti,ab,kw OR tumour*:ti,ab,kw OR malignan*:ti,ab,kw OR carcinoma*:ti,ab,kw OR adenocarcinoma*:ti,ab,kw)) | **38735** |
|  |  |  |
| 3 | #1 AND #2 | **61** |

Supplementary table 2. A distinct pattern of microbial profile in triple negative breast cancer patients.

| **Viral Agent** | **Bacterial Agent** | **Fungal Agent** |
| --- | --- | --- |
| *Human herpesvirus 5/HCMV* | *Brevundimonas* | *Piedraia* |
| *Human herpesvirus 8/KSHV* | *Mobiluncus* | *Phialophora* |
| *Simian virus 40* | *Geobacillus* | *Fonsecaea* |
| *Hepatitis C virus genotype 1* | *Propionibacterium* | *Pleistophora* |
| *Human T-lymphotropic virus 2* | *Actinomyces* | *Paecilomyces* |
| *Orf virus* | *Arcanobacterium* |  |
| *Pseudocowpox virus* | *Peptoniphilus* | **Parasitic Agent** |
| *Human herpesvirus 4/EBV* | *Sphingobacterium* | *Trichuris* |
| *Bovine papular stomatitis virus* | *Prevotella* | *Toxocara* |
| *Okra mosaic virus* | *Providencia* | *Thelazia* |
| *Human papillomavirus 2* | *Escherichia* | *Babesia* |
| *Human T-lymphotropic virus 1* | *Rothia* | *Leishmania* |
| *Hepatitis B virus* | *Brucella* |  |
| *Human herpesvirus 1* | *Capnocytophaga* |  |
| *Human papillomavirus type 16* |  |  |
| *Moloney murine leukemia virus* |  |  |
| *Merkel cell polyomavirus* |  |  |
| *Mouse mammary tumor virus* |  |  |
| *Human papillomavirus type 6b* |  |  |
| *Human papillomavirus 18* |  |  |
| *JC polyomavirus* |  |  |
| *Hepatitis GB virus A* |  |  |
| *Fujinami sarcoma virus* |  |  |
